# Synthesis of Nitroaromatic Compounds via Three-Component Ring Transformations

**DOI:** 10.3390/molecules26030639

**Published:** 2021-01-26

**Authors:** Song Thi Le, Haruyasu Asahara, Nagatoshi Nishiwaki

**Affiliations:** 1 School of Environmental Science and Engineering, Kochi University of Technology, Tosayamada, Kami, Kochi 782-8502, Japan; lesong1986@gmail.com (S.T.L.); asahara@phs.osaka-u.ac.jp (H.A.); 2 Center for Equipment and Labour Safety, Vietnam Institute for Building Materials (VIBM), Ministry of Construction, 235 Nguyen Trai, Thanh Xuan, Hanoi 100000, Vietnam; 3 Research Center for Molecular Design, Kochi University of Technology, Tosayamada, Kami, Kochi 782-8502, Japan; 4 Graduate School of Pharmaceutical Sciences, Osaka University, Yamadaoka 1-6, Suita, Osaka 565-0871, Japan

**Keywords:** dinitropyridone, three-component ring transformation, nitropyridine, nitroaniline, bicyclic intermediate, nitromalonaldehyde

## Abstract

1-Methyl-3,5-dinitro-2-pyridone serves as an excellent substrate for nucleophilic-type ring transformation because of the electron deficiency and presence of a good leaving group. In this review, we focus on the three-component ring transformation (TCRT) of dinitropyridone involving a ketone and a nitrogen source. When dinitropyridone is allowed to react with a ketone in the presence of ammonia, TCRT proceeds to afford nitropyridines that are not easily produced by alternative procedures. Ammonium acetate can be used as a nitrogen source instead of ammonia to undergo the TCRT, leading to nitroanilines in addition to nitropyridines. In these reactions, dinitropyridone serves as a safe synthetic equivalent of unstable nitromalonaldehyde.

## 1. Introduction

### 1.1. Ring Transformation

**Scheme 1:**
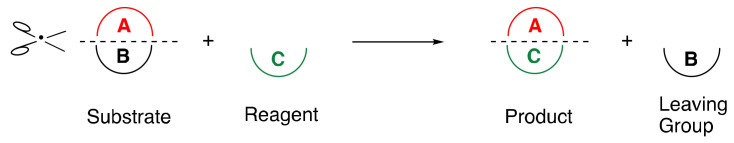
General concept of the ring transformation.

Ring transformation is a powerful synthetic method that accompanies the “scrap and build” of cyclic compounds. The general concept of this method is shown in Scheme 1. When a substrate (**A** + **B**) is reacted with a reagent (**C**), the partial structure (**A**) of the substrate is transferred to the reagent to construct a new ring system (**A** + **C**), simultaneously eliminating the leaving group (**B**). This reaction facilitates the synthesis of functionalized compounds that are not easily afforded by alternative procedures. 

**Scheme 2:**
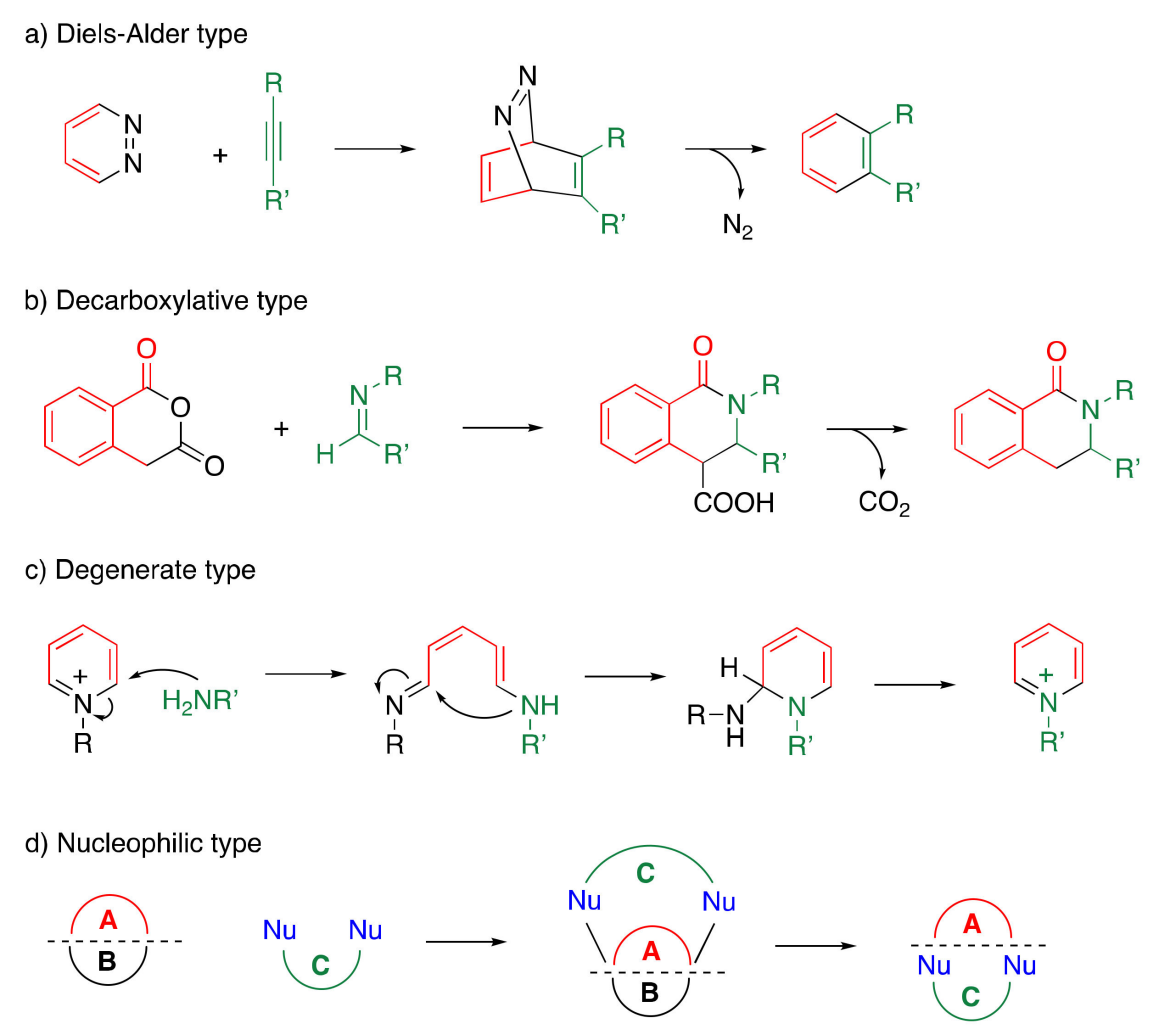
General concepts of four kinds of ring transformations.

There are four types of ring transformations, namely, Diels–Alder-type, decarboxylative, degenerate, and nucleophilic-type ring transformations (Scheme 2). The most commonly used methods are Diels–Alder-type ring transformation (type a) [1,2,3] and decarboxylative ring transformation (type b) [4,5,6], wherein the substrates have a good leaving group as a partial structure (molecular nitrogen and carbon dioxide, respectively). Degenerated ring transformation was energetically studied by van der Plas [7]. This reaction proceeds through the addition of nucleophile–ring opening–ring closure (ANRORC) mechanism. The nucleophilic-type ring transformation has not been studied extensively as compared to the other three ring transformations [8,9,10,11,12,13].

### 1.2. Suitable Substrate for Nucleophilic-Type Ring Transformation

**Figure 1:**
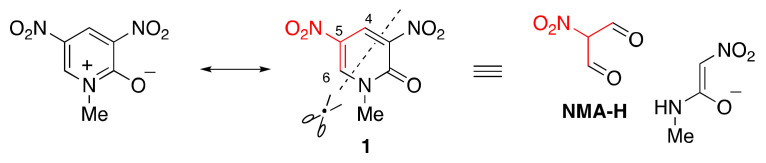
Resonance structure of dinitropyridone **1** and its partial structure.

To cause the nucleophilic-type ring transformation, a substrate requires three conditions: (1) high electron deficiency, (2) low aromatic stabilizing energy, and (3) the presence of a good leaving group as the partial structure. Based on these considerations, 1-methyl-3,5-dinitro-2-pyridone (**1**) appears to be a suitable structure for this purpose (Figure 1). The electron-withdrawing nitro and carbonyl groups, besides the ring nitrogen atoms, diminish the electron density of this compound. As shown in the resonance form, though pyridone **1** exhibits aromaticity, it is easily destroyed because of the minimal contribution of the betaine resonance structure. In addition, the partial structure can be easily eliminated as a stable anion of nitroacetamide. When the ring transformation proceeds at the 4- and 6-positions accompanied by elimination of anionic nitroacetamide, the C4–C5–C6 moiety of pyridone **1** serves as the synthetic equivalent of nitromalonaldehyde (**NMA-H**). **NMA-H** is typically considered a synthon in retrosynthesis. However, **NMA-H** is too unstable to be isolated. Instead, its sodium salt (**NMA-Na**) has been widely used, although it should be handled carefully because of the explosive impurities [14]. Thus, it is necessary to develop a safe synthetic equivalent of **NMA-H** [15]. From this perspective, a nucleophilic-type ring transformation using pyridone **1** is a useful synthetic method for versatile nitro compounds because of its higher safety. 

**Scheme 3:**
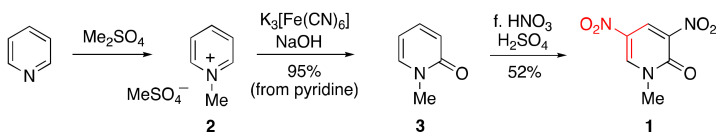
Preparation of 1-methyl-3,5-dinitro-2-pyridone (**1**).

Dinitropyridone **1** can be easily prepared from pyridine in three steps. After the conversion of pyridine to *N*-methylpyridinium salt **2** by dimethyl sulfate, oxidation with ferricyanide under alkaline conditions in one pot leads to the formation of 1-methyl-2-pyridone **3**. The subsequent nitration of **3** by fuming nitric acid with sulfuric acid forms dinitropyridone **1** (Scheme 3).

## 2. Ring Transformation of 1 with Carbon Dinucleophiles

### 2.1. Aminolysis of Dinitropyridone **1**

**Scheme 4:**
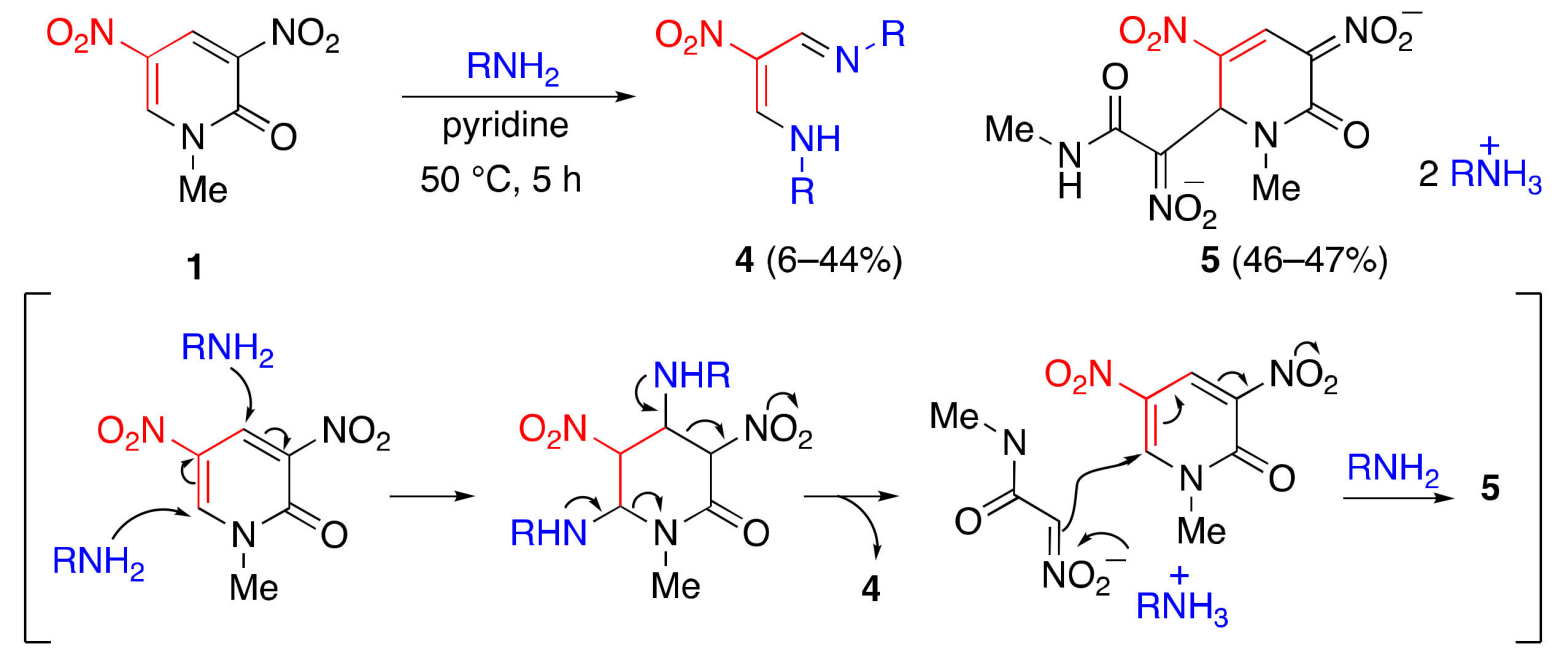
Aminolysis of dinitropyridone **1** and a plausible mechanism for the aminolysis of **1**.

Dinitropyridone **1** serves as a suitable substrate for nucleophilic-type ring transformation, which can be confirmed through aminolysis. The ring opening reaction of **1** proceeds upon treatment with amine, leading to nitro-substituted azadienamine **4** and dianionic product **5** (Scheme 4) [16]. The latter is formed by the addition of anionic nitroacetamide to pyridone **1**. This reaction is initiated by the addition of amines at the 4- and 6-positions. The subsequent cleavage of two C–C bonds furnishes azadienamine **4**, which indicates that anionic nitroacetamide serves as a good leaving group. However, it also serves as a nucleophile to form adduct **5** (Scheme 4). 

**Scheme 5:**
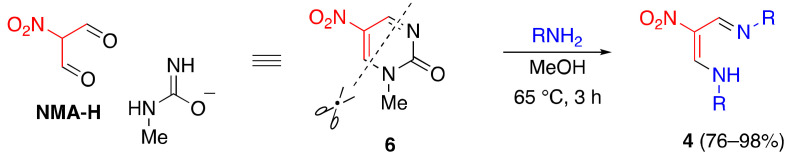
Aminolysis of nitropyrimidinone **6**.

Azadienamine **4** can be used as an excellent ligand to form diverse metal complexes [17,18,19]. From the perspective of ligand preparation, this reaction is not suitable, as dinitropyridone **1** is consumed by eliminated nitroacetamide. This problem is overcome by using 1-methyl-5-nitro-2-pyrimidinone (**6**) instead of dinitropyridone **1**, as the eliminated urea is less nucleophilic than nitroacetamide and can thus avoid the consumption of **6** (Scheme 5) [20].

### 2.2. Reaction of Dinitropyridone **1** with 1,3-dicarbonyl Compounds

The landmark work on nucleophilic-type ring transformation was achieved by Matsumura et al. (Table 1) [21,22]. When dinitropyridone **1** is allowed to react with sodium enolate of diethyl acetonedicarboxylate **7a**, the ring transformation can afford a high yield of 2,6-difunctionalized 4-nitrophenol **8a**. This reaction can be applied to reagents **7b**–**d**, each possessing one active methylene group, to afford the corresponding nitrophenols **8b**–**d**.

**Scheme 6:**
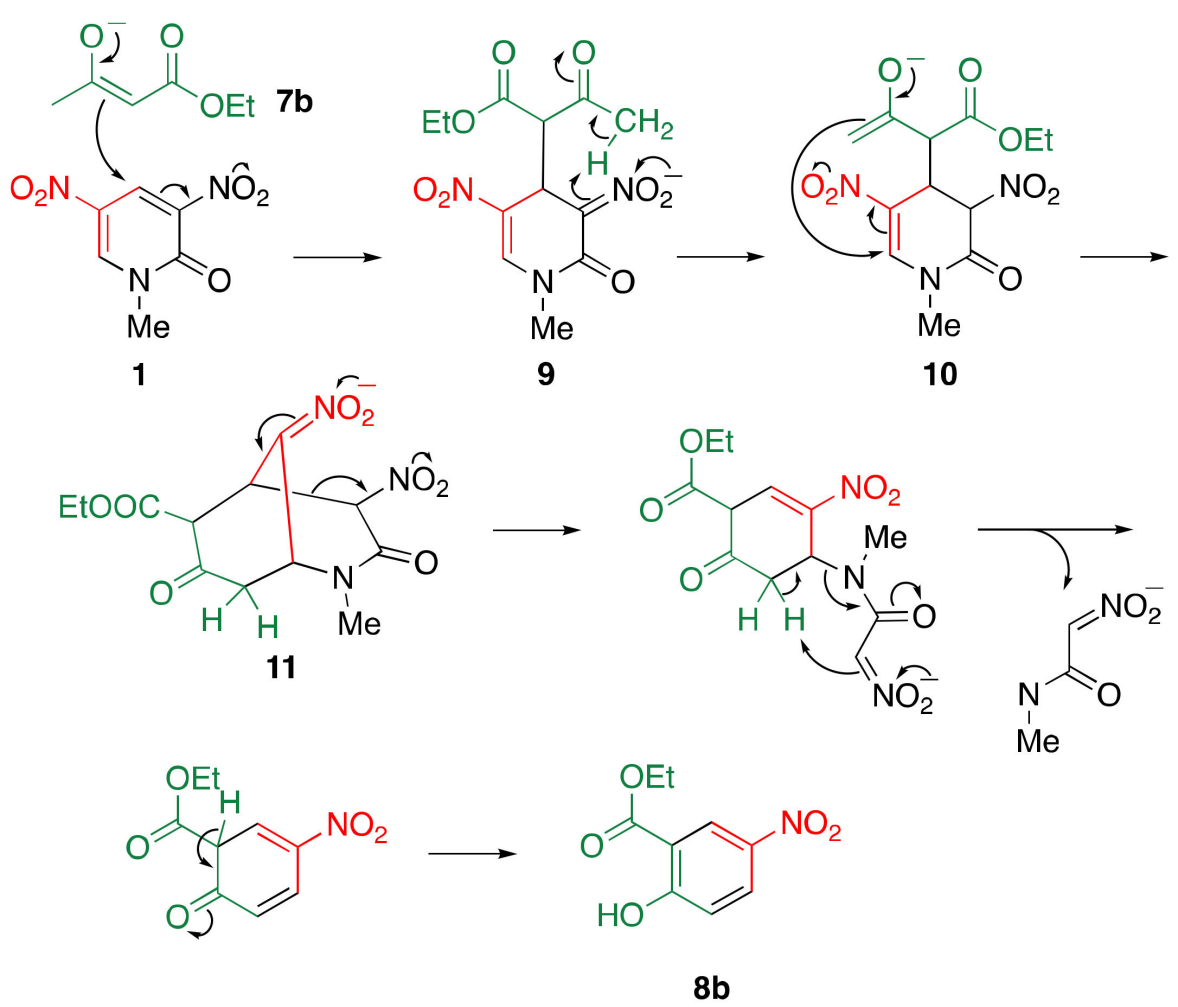
A plausible mechanism for the formation of **8**.

**Scheme 7:**
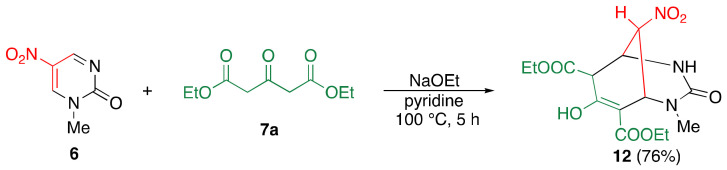
Synthesis of bicyclic compound **12**.

A plausible mechanism for this reaction is illustrated in Scheme 6. The enolate ion **7b** attacks the 4-position of pyridone **1** to afford adduct intermediate **9**, and the regenerated enolate **10** attacks the 6-position of **1**, leading to bicyclic intermediate **11**, from which the stable anionic nitroacetamide is eliminated to furnish nitrophenol **8b**; the bicyclic intermediate **11** can be isolated from the reaction mixture [21]. In addition, the reaction of nitropyrimidinone **6** and diethyl acetonedicarboxylate **7a** also affords bicyclic product **12** in high yield because unstable anionic urea cannot eliminate [23] (Scheme 7). Based on these results, the ring transformation is considered to proceed via bicyclic intermediates.

## 3. Three-Component Ring Transformation (TCRT)

### 3.1. General Concept of TCRT

**Scheme 8:**
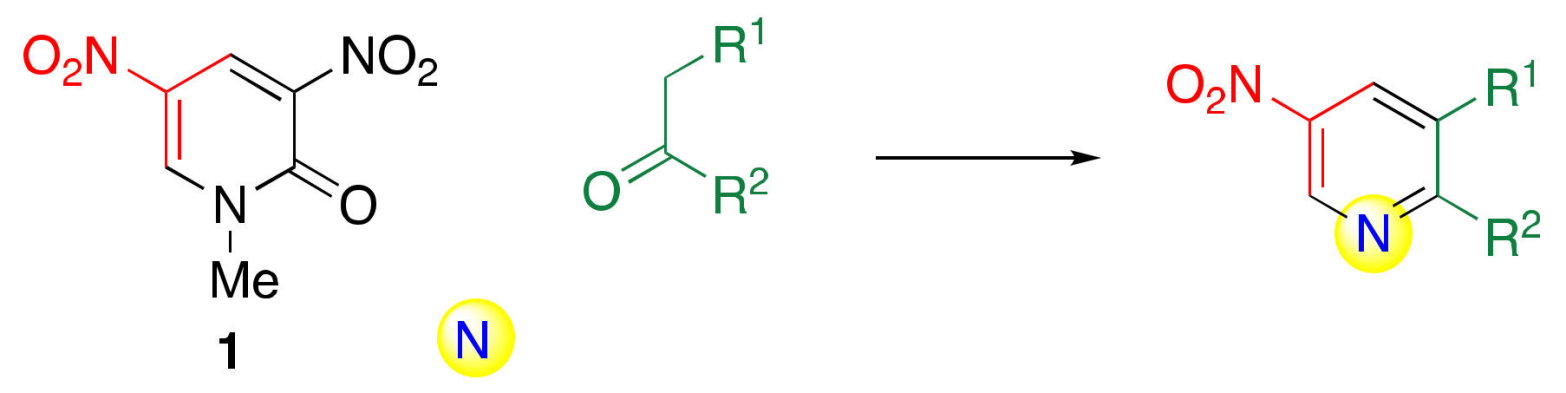
The general concept of TCRT.

As mentioned previously, dinitropyridone **1** is highly reactive when used as the substrate in the nucleophilic-type ring transformation. The 1,3-dicarbonyl compounds **7** are excellent dinucleophilic reagents. However, the diversity of the available 1,3-dicarbonyl compounds **7** is low, which only affords few products **8**. If simple ketones can be used instead of **7**, the synthetic utility of the ring transformation should be improved. In such cases, it is necessary to use a nitrogen source as ketone is a mononucleophilic reagent. This process is referred to as three-component ring transformation (TCRT) (Scheme 8).

### 3.2. TCRT Using Ammonia as the Nitrogen Source

Tohda et al. studied the reaction of dinitropyridone **1** with ketones in the presence of ammonia (Table 2) [24]. When a methanol solution of pyridone **1** is heated with cyclohexanone **13a** in the presence of ammonia (20 equiv.) at 70 °C (condition A), cyclohexa[*b*]pyridine **14a** is obtained in 83% yield. However, this method suffers from the narrow scope of ketones. The TCRT using cyclopentanone **13b** under the same conditions forms cyclopenta[*b*]pyridine **14b** in a considerably lower yield. When acetophenone **15a** is allowed to react under the same conditions, TCRT proceeds similarly; however, the yield is low owing to the competitive ammonolysis of substrate **1**. To overcome this disadvantage, it is important to employ severe conditions (heating with larger amounts of ammonia (140 equiv.) at 120 °C in an autoclave (condition B)). This reaction is applicable to other aromatic ketones **15b**–**h** to afford the corresponding 2-(het)aryl-5-nitropyridines **16b**–**h**, respectively. The ketone is not required to have an acetyl group, and propiophenone **15i** undergoes the TCRT, leading to trisubstituted pyridine **16i**. In the case of aromatic ketones **15a**–**i**, employment of condition B is effective for obtaining pyridines **16a**–**i** in better yields. In contrast, ketone **15j** possessing an α‘-proton forms pyridine **16j** with better yield under condition A, as severe conditions cause side reactions. Indeed, pinacolone **15k** without an α‘-proton undergoes the TCRT more efficiently.

This TCRT efficiently proceeds under mild conditions (condition A) only when cyclohexanone **13a** is used as the reagent. In other words, this protocol is an effective approach to [*b*]-fused 5-nitropyridines. This reaction is often employed for synthesizing biologically active compounds, medicines, and their synthetic intermediates.

**Figure 2:**
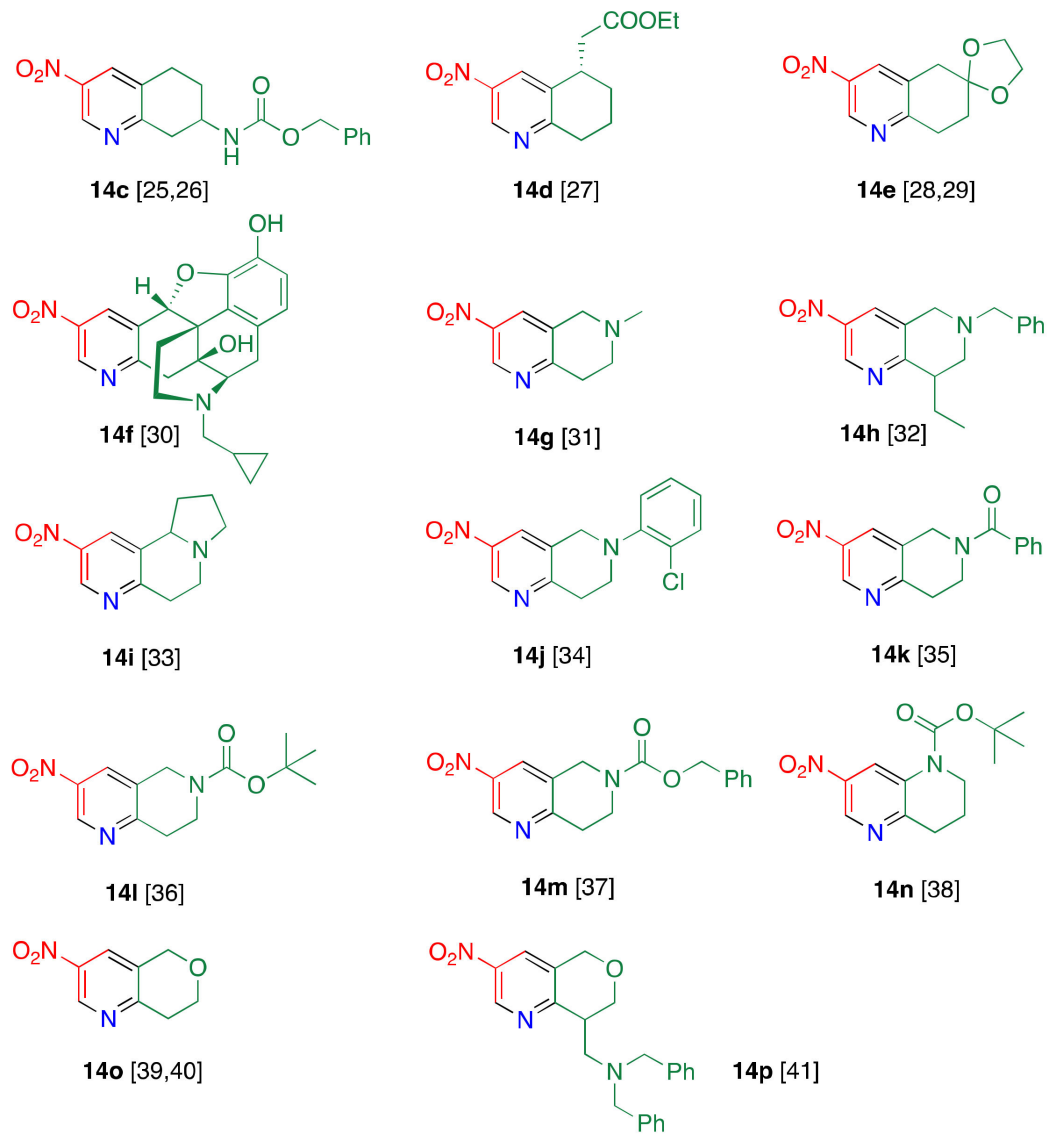
Nitropyridines condensed with a six-membered ring.

Cyclohexa[*b*]pyridines **14c**–**f** (Figure 2) are synthesized by TCRT using ammonia as a nitrogen source, in which functional groups such as carbamate, ester, and acetal are tolerated during the reaction [25,26,27,28,29,30]. Notably, multiple functionalities remain during the TCRT to afford a complex structure **14f**. Piperidine-4-ones are usable as reagents in TCRT to produce 5,6,7,8-tetrahydro-1,6-naphthyridines **14g**–**m** [31,32,33,34,35,36,37]. Not only *N*-alkylated derivatives **14g**–**i**, but also *N*-aryl derivative **14j** and *N*-acyl derivatives **14k**–**m** are available. When unsymmetrical pieridine-3-one is used, two condensed pyridines are formed, including 1,5-naphthyridine **14n** [38]. Tetrahydropyran-4-one can be used for this method, which makes pyranopyridines **14o** and **14p** available [39,40,41]. 

**Figure 3:**
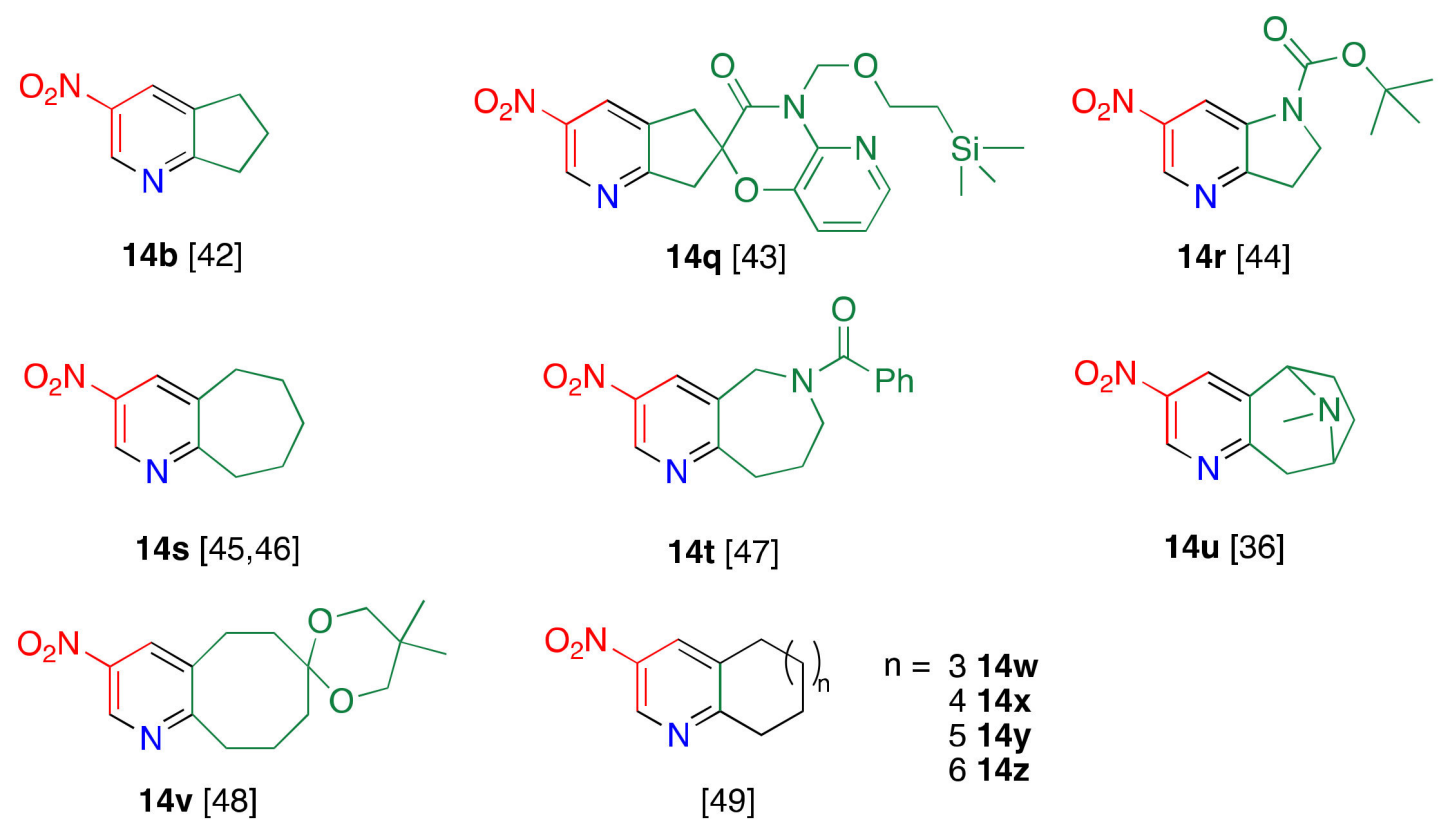
Nitropyridines condensed with a five-membered ring and with a larger ring.

Cycloalkanones with different ring sizes can also be used as reagents in this TCRT (Figure 3). Cyclopenta[*b*]pyridine **14q**, even though it has a complex structure, can be synthesized by altering the cyclopentanone to the corresponding one [42,43]. When pyrrolidine-3-one is used, 4-azaindole **14r** is obtained [44].

Furthermore, cyclohepta[*b*]pyridine **14s** can be synthesized upon treatment of pyridone **1** with cycloheptanone **13s** [45,46]. When aza-containing cycloheptanone **13t** and bridged cycloheptanone **13u** are employed, cycloheptapyridine **14t** [47] and tricyclic pyridines **14u** [36] are formed. Nitropyridines condensed with a larger ring (from eight to eleven membered rings) can be prepared by only altering cycloalkanones [48,49].

### 3.3. Reaction Mechanism of TCRT

**Scheme 9:**
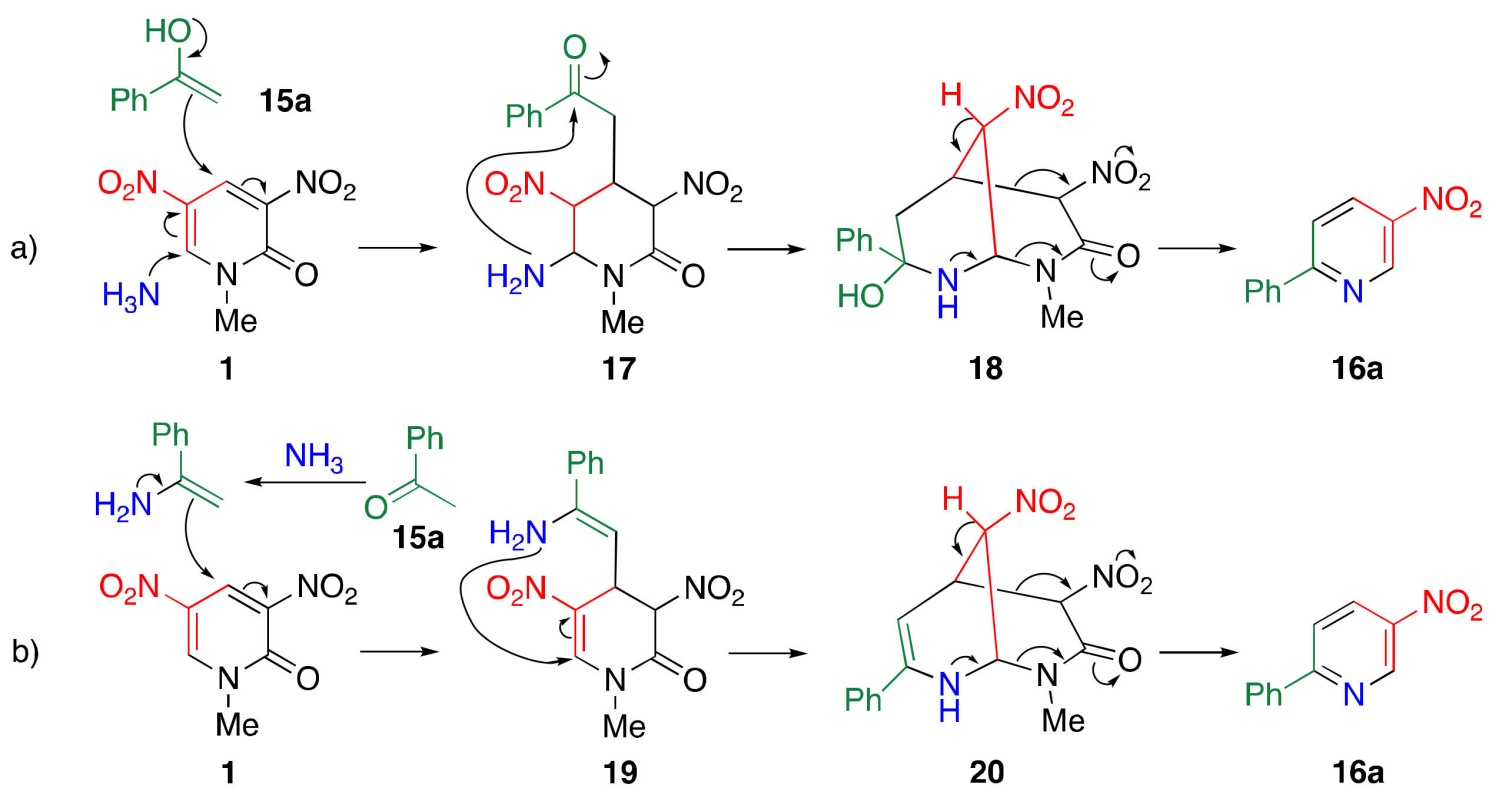
Plausible mechanism of the TCRT. (**a**) Including attacks of two nucleophiles to pyridone **1**. (**b**) Including an attack of enamine preformed in situ.

Two plausible mechanisms of TCRT are illustrated in Scheme 9. As mentioned in Section 2.1, both the 4- and 6-positions of dinitropyridone **1** are highly electrophilic, and are thus attacked by the enol form of **15a** and ammonia to form adduct intermediate **17** (path a) [24]. The same product, **16a**, is obtained when the ammonia and enol switch positions to attack. The amino group intramolecularly attacks the carbonyl group derived from **15a**, leading to bicyclic intermediate **18**, from which nitroacetamide is eliminated and accompanied by aromatization to afford nitropyridine **16a**. Another possibility is that ketones are converted to enamines, which might serve as an actual nucleophile (path b) [50]. After adding the enamine to pyridone **1**, the amino group intramolecularly attacks the 6-position to form bicyclic intermediate **20**, and elimination of nitroacetamide leads to the formation of nitropyridine **16a**. 

### 3.4. TCRT Using Ammonium Acetate as the Nitrogen Source

This TCRT proceeds efficiently when reactive cycloalkanones **13** are employed as reagents. In other words, when less reactive ketones such as **15a** are used, both electrophilic sites of **1** are attacked by ammonia, which undergoes ammonolysis to consume pyridone **1** competitively. Le et al. mitigated this problem by using a less nucleophilic ammonium acetate as a nitrogen source instead of ammonia. 

When pyridone **1** is reacted with acetophenone **15a** and three equivalents of ammonium acetate, nitropyridine **16a** and a bicyclic product **21a** are obtained (Table 3) [51]. The former is produced by TCRT, and the latter is formed by the insertion of **15a** and nitrogen between the N1 and C2 positions of pyridone **1**. Isolated **21a** can be converted to **16a** upon treatment with ammonium acetate, which indicates that there is equilibrium between these products. Thus, **16a** is a thermodynamically controlled product, and **21a** is a kinetically controlled product. The ratio of **16a** increases as larger amounts of ammonium acetate or microwave heating are used. The use of larger amounts of ammonium acetate prolongs the actual reaction time, because it decomposes to gaseous ammonia and acetic acid upon heating. 

**Scheme 10:**
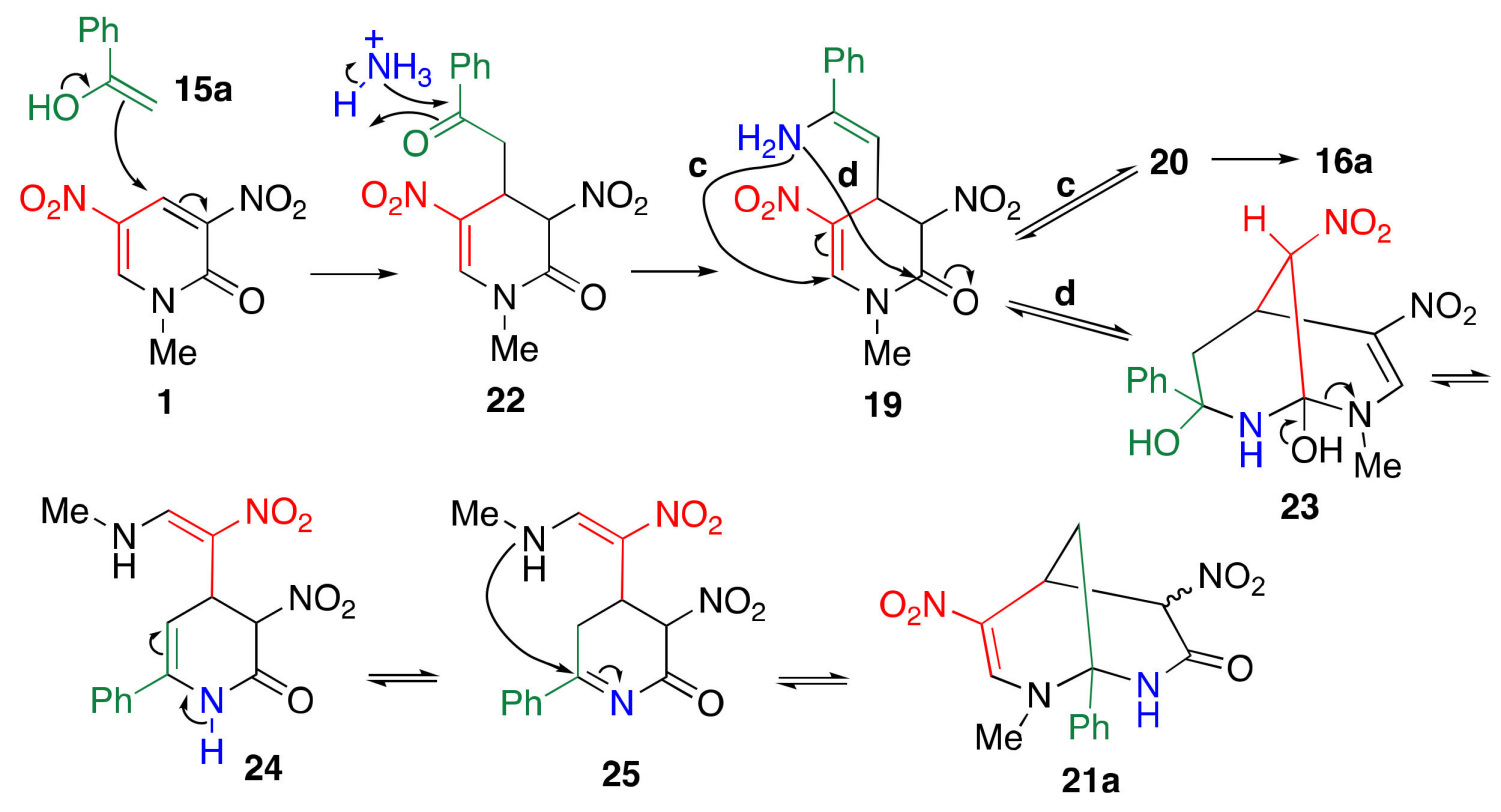
A plausible mechanism for the formation of bicyclic product **21a**.

The formation of bicyclic product **21a** is considered to proceed as shown in Scheme 10. After addition of an enol form of **15a** to the 4-position of **1**, the acyl moiety of **22** is converted to enamine **19** by the ammonium ion. When the amino group of **19** intramolecularly attacks at the 6-position (path c), nitropyridine **16a** is formed via bicyclic intermediate **20**, as illustrated in Scheme 9. In contrast, the amino group of **19** attacks the carbonyl group, and degenerated ring transformation proceeds to afford **24**. After prototropy leading to **25**, the methylamino group attacks the imino functionality to afford bicyclic product **21a**. However, the aminal structure of **21a** is easily cleaved under acidic conditions to regenerate intermediate **19**, which furnishes aromatized product **16a**, predominantly under severe conditions.

This method is applicable to other aromatic ketones **15a**–**q** (Table 4). TCRT efficiently proceeds in reactions using both electron-rich and electron-poor ketones, among which electron-poor ketones reveal lower reactivity and require larger amounts of ammonium acetate (longer reaction time). In cases of electron-poor ketones **15e**, **15f**, and **15o**, bicyclic products **21e**, **21f**, and **21o** are obtained, respectively. The ketone is not required to have an acetyl group, and ketones **15i** and **15q** afforded the corresponding trisubstituted pyridines **16i** and **16q** in almost quantitative yields, respectively.

α,β-Unsaturated ketones **26** and **28** can also be used for the TCRT (Table 5 and Table 6) [52]. These ketones are less reactive, requiring 15–30 equivalents of ammonium acetate. Among the three styryl ketones, electron-rich ketone **26b** reveals higher reactivity, which facilitates the approach to electron-deficient pyridone **1**. The reaction with alkynyl ketones **28** efficiently furnishes alkynylpyridines **29**. When silylethynyl ketone **28c** is used, the desilylated product **29d** is also obtained. 

For the C–C bond formation on the pyridine framework, the Heck, Suzuki, Stille, and Sonogashira reactions are commonly used. However, these methods require the use of poisonous and expensive transition metals and a purification step to avoid metal contamination of the products. In addition, troublesome multistep reactions are necessary to prepare the substrates for these reactions (2-halo-5-nitropyridines). Thus, the TCRT is a metal-free supplementary method for the abovementioned reactions.

### 3.5. Preparation of 3-substituted 5-nitropyridines **31** by TCRT

When dinitropyridone **1** is allowed to react with aldehyde **30** and ammonia as a nitrogen source, TCRT does not occur at all. In such a case, the use of ammonia/ammonium acetate as a mixed nitrogen source is effective to undergo the TCRT. However, the yields of **31** are low, as highly reactive aldehyde **30** causes side reactions such as self-condensation [24,53]. Using only ammonium acetate helps the TCRT to afford the corresponding pyridines **31a**–**f** in moderate to high yields (Table 7) [54]. This protocol facilitates the introduction of not only a bulky alkyl group such as a *tert*-butyl but also an aromatic group into the pyridine framework with simple experimental manipulations. 

### 3.6. TCRT Using Cyclic Ketones **13**

**Scheme 11:**
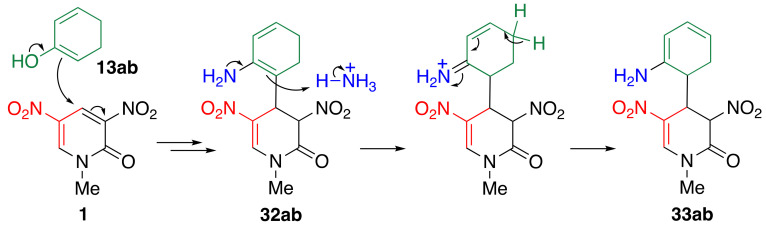
A plausible mechanism for the double bond migration.

Dinitropyridone **1** undergoes TCRT with cycloalkanone **13** in the presence of ammonium acetate, leading to cycloalka[*b*]pyridines **14** (Table 8) [55]. Cycloalkanones **13** with various ring sizes efficiently react under conventional heating (Condition C) to afford the corresponding nitropyridines condensed with five-, six-, seven-, and eight-membered rings. The reaction time is considerably shortened by using microwave heating (Condition D). In this reaction, the unsymmetrical ketone, 2-methylcyclohexanone **13aa**, which reacts at the 6-position not at the 2-position, as aromatization is prevented by a methyl group in the latter case, can also be used as a reagent. When 2-cyclohexenone **13ab** is used, migration of the double bond is observed, which may occur after the addition of ketone **13ab** to pyridone **1** and the subsequent conversion to dienamine **32ab**, leading to the formation of dienamine **33ab** (Scheme 11).

### 3.7. Reconsideration about the Reaction Mechanism of TCRT

**Scheme 12:**
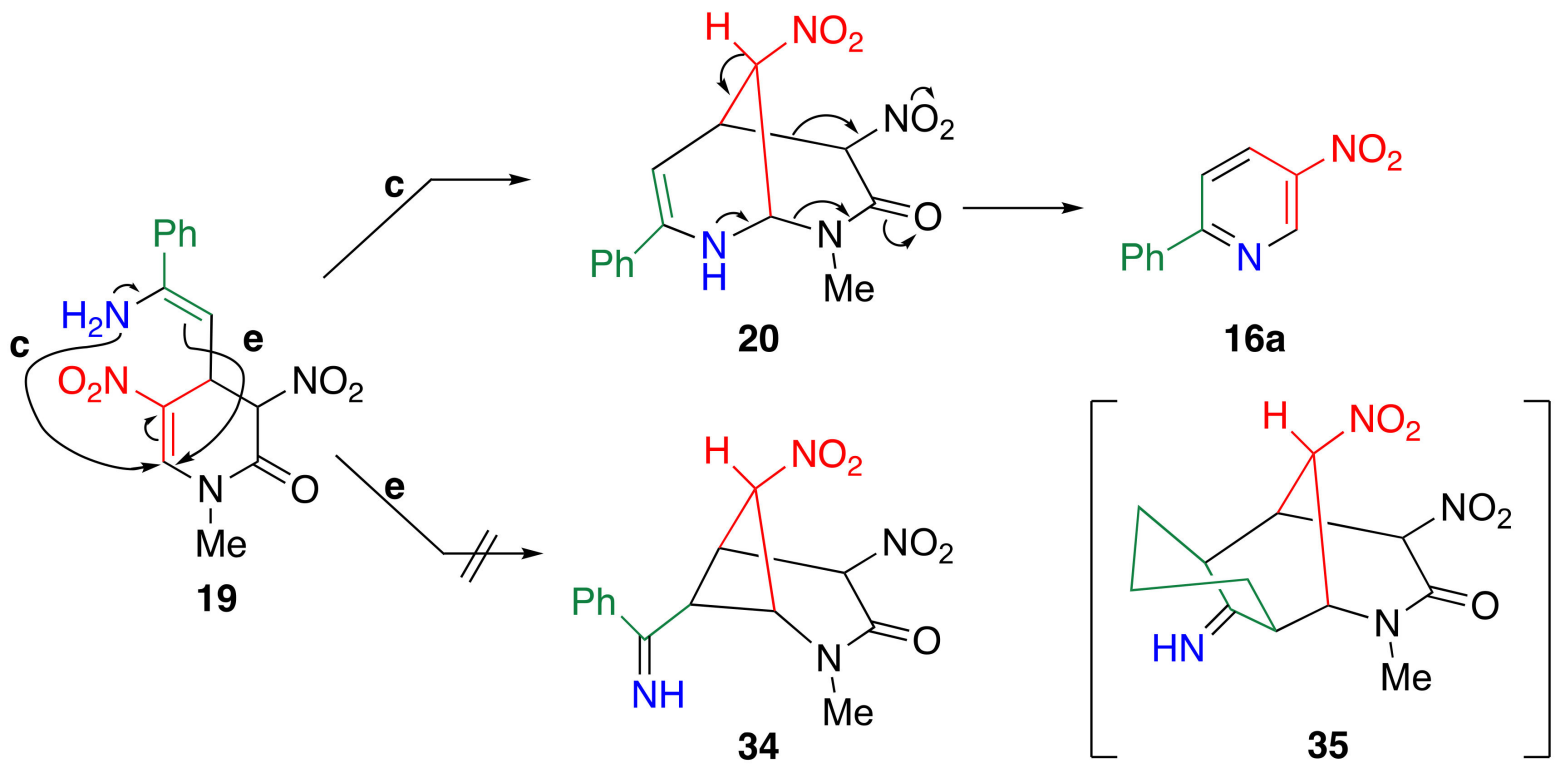
Plausible mechanism using aromatic ketone **15** and cycloalkanone **13**.

As shown in Scheme 10, the TCRT is initiated by the addition of the enol form of a ketone to the 4-position of dinitropyridone **1**, after which the acyl group of adduct **19** is converted to enamine **20** by the ammonium ion. Enamine has an ambident property, where β-carbon is generally more nucleophilic than the amino group. In the case of adduct intermediate **19** derived from aromatic ketone **15**, *N*-attack (path c) forms a six-membered ring to afford bicyclic intermediate **20**, from which nitropyridine **16** is obtained, accompanied by the elimination of nitroacetamide (Scheme 12). In contrast, if a *C*-attack (path e) occurs, sterically strained four-membered ring **34** is formed. Hence, nitropyridine **16** is formed as the sole product in this TCRT. In cases of α,β-unsaturated ketones **26** and **28** and aldehydes **30**, a similar reactivity is observed, as these carbonyl compounds have only one kind of α-hydrogen.

**Scheme 13:**
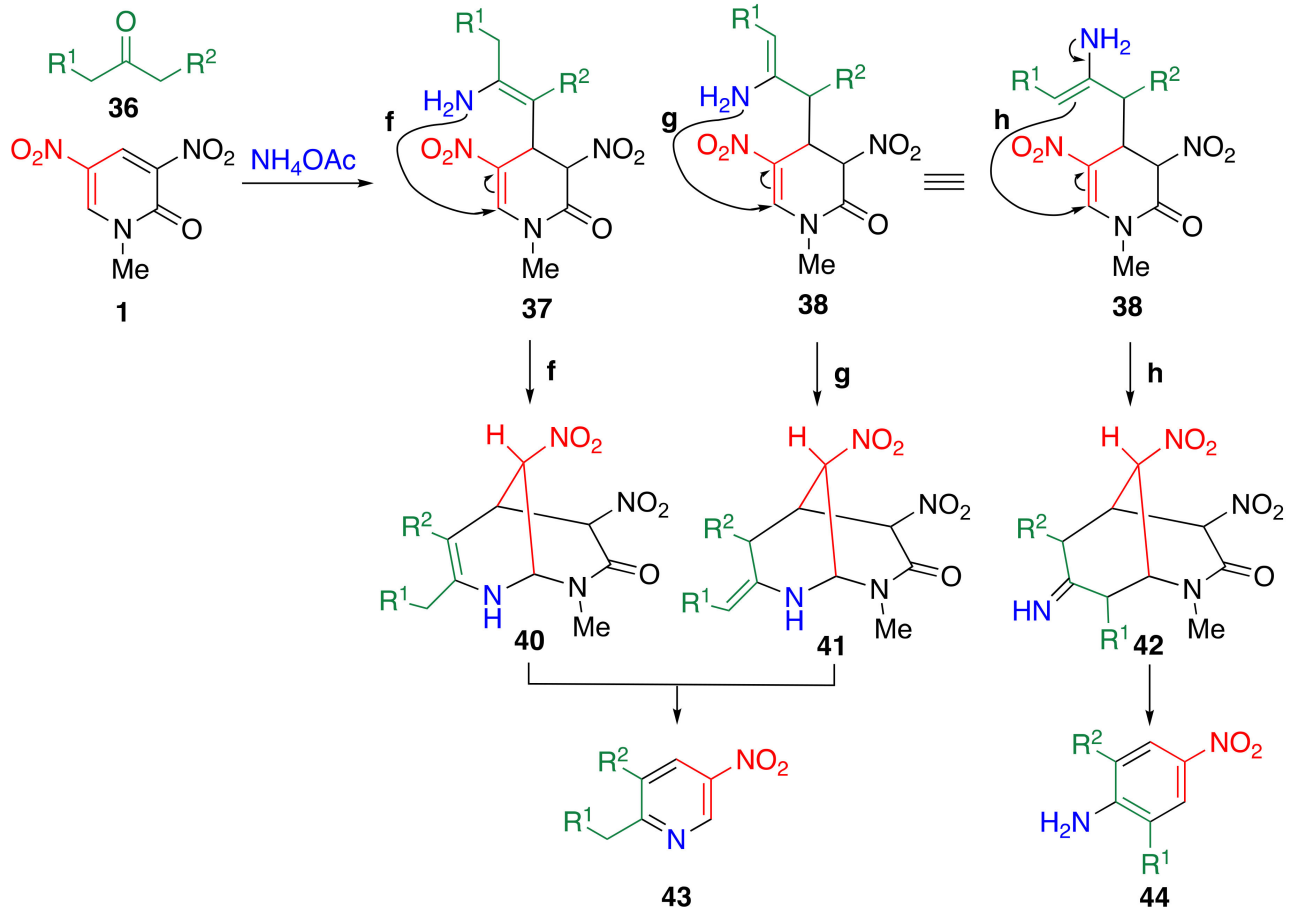
Plausible mechanisms of TCRT when an aliphatic ketone **36** is employed as a reagent.

In the case of aliphatic ketones **36**, two types of enamines (**37** and **38**) are possibly formed (Scheme 13). While the intermediate **37** cannot cause a *C*-attack similar to **19**, the intermediate **38** can cause both *N*- and *C*-attacks to furnish bicyclic intermediates **41** and **42**, respectively. From bicyclic intermediates **40** and **41**, nitropyridine **43** is formed. In contrast, 2,6-disubstituted 4-nitroaniline **44** should form when nitroacetamide is eliminated from bicyclic intermediate **42**. Thus, two ring-transformed products (**43** and **44**) are yielded when aliphatic ketones **36** are used as reagents.

In the reactions of pyridone **1** with cycloalkanone **13**, only nitropyridine **14** is formed (Table 8). Although the adduct of **1** and cycloalkanone **13** can form two kinds of enamines, one enamine can form a six-membered ring as a result of C-attack, and the formed intermediate **35** is too strained to be formed (Scheme 12). 

### 3.8. TCRT Using Aliphatic Ketones **36**

When dinitropyridone **1** is subjected to a reaction with aliphatic ketones **36** in the presence of ammonium acetate, two types of TCRT occur to afford nitropyridines **43** and nitroanilines **44** (Table 9) [56]. Generally, 2,6-disubstituted 4-nitroanilines **44** are prepared from the corresponding anilines by nitration under harsh reaction conditions, wherein protection and deprotection of the amino groups are necessary [57]. Furthermore, the preparation of this compound suffers from the limitation of Friedel–Crafts alkylation. There are several limitations for the Friedel–Crafts alkylation, such as the following: (1) The monoalkylated product undergoes further alkylation, (2) it is difficult to introduce two different alkyl groups, (3) primary alkyl groups longer than the ethyl group cannot be introduced, (4) a phenyl group cannot be introduced, and (5) nitrobenzene and aniline do not facilitate the alkylation. The TCRT overcomes these disadvantages.

When dinitropyridone **1** is reacted with 3-pentanone in the presence of five equivalents of ammonium acetate, nitroaniline **44a** and nitropyridine **43a** are obtained at 50% and 44%, respectively, resulting from two types of TCRT. In contrast, the ratio of **44a** to **43a** increases significantly without a decrease in total yield, indicating the presence of an equilibrium between bicyclic intermediates **42** and **41** (Scheme 13). The substituents can be modified by altering only the ketones **36** (Table 9). Monoalkylated nitroanilines **44c**–**e** and unsymmetrical nitroanilines **44h** and **44i** are available from the corresponding unsymmetrical ketones **36**. Furthermore, it is easy to prepare nitroanilines **44g**–**i** possessing a propyl or phenyl group, which cannot be introduced by the Friedel–Crafts reaction. However, steric repulsion by the phenyl groups prevents the formation of bicyclic intermediate **42i**. 

A combination of propylamine **45A** and acetic acid can be used as a reagent instead of ammonium acetate, which facilitates *N*-modification of the amino group as well as the benzene ring of nitroaniline **46** (Table 10). This method is applicable to secondary amines, pyrrolidine **45B** and diethylamine **45C**, to afford *N,N*,2,6-tetrasubstituted 4-nitroanilines **46B** and **46C**, respectively. This reaction also enables the introduction of a propyl or phenyl group into the benzene framework, which cannot be introduced by the Friedel–Crafts reaction.

As shown in Scheme 13, the TCRT proceeds through the *C*-attack of the intermediately formed enamine **38**. This means that functionalized nitoanilines **48** can be prepared if a similar structure is available via an alternative route. For this purpose, relatively stable enaminones **47** prepared from 1,3-dicarbonyl compounds **7** and amine **45** are considered suitable. When dinitropyridone **1** reacts with enaminone **47**, nucleophilic-type ring transformation proceeds to afford 2-functionalized 4-nitroaniline **48** (Table 11) [58]. This protocol facilitates the modification of the functional group and amino group of **48** by altering 1,3-dicarbonyl compounds **7** and amine **45**. Diketones **7c** and **7e** as well as keto easter **7b** can be used as 1,3-dicarbonyl compounds. These reagents are not required to possess an acetyl group (R^1^ = H), and **7f** undergoes similar ring transformations. Bulky amines such as *tert*-butylamines **45D** and **45E** and less nucleophilic anilines **45F** and **45G** can be used as amines. Even though amines have a functional group, the corresponding nitroaniline **48Hb** is obtained. Furthermore, cyclic and acyclic secondary amines **45B** and **45C** can be used for this reaction, which results in 2-functionalized *N,N*-dialkyl-4-nitroanilines **48Bc**, **48Ca**, and **48Ce**.

## 4. Conclusions 

When dinitropyridone **1** is subjected to a reaction with cycloalkanones **13** in the presence of ammonia, nucleophilic-type TCRT efficiently proceeds to afford nitrated cycloalka[*b*]pyridines **14**. In this reaction, pyridone **1** serves as a synthetic equivalent of unstable **NMA-H**. However, this method is applicable only to cycloalkanones **13**, as the competitive ammonolysis of **1** cannot be ignored in cases of other types of ketones.

This disadvantage is overcome by using the less nucleophilic ammonium acetate as a nitrogen source instead of ammonia. Aromatic ketones **15**, alkenyl ketones **26**, alkynyl ketones **28**, and aldehyde **30** undergo TCRT to furnish the corresponding pyridines that are not easily available by alternative methods, including transition-metal-catalyzed coupling reactions. When acyclic aliphatic ketones **36** are used as the reagent, the TCRT proceeds in different modes to give 4-nitroaniline derivatives **44**. In this reaction, a combination of amine and acetic acid is usable, leading to the synthesis of *N,N*,2,6-tetrasubstituted 4-nitroanilines **46**. Furthermore, functionalized nitroanilines **48** are available using enaminones **47** as a reagent. 

In addition to the easy modification of the product framework, the reaction is conducted under mild conditions with simple experimental manipulations, which are more practical. These features facilitate the construction of a library of compounds that are not easily available by other methods. In particular, compounds possessing both electron-donating and electron-withdrawing groups (push–pull systems) are necessary for developing novel functional materials such as medicines, agrochemicals, and non-linear optical materials. Therefore, the TCRT will provide a new synthetic tool for researchers studying in this field.

## Figures and Tables

**Table 1:**
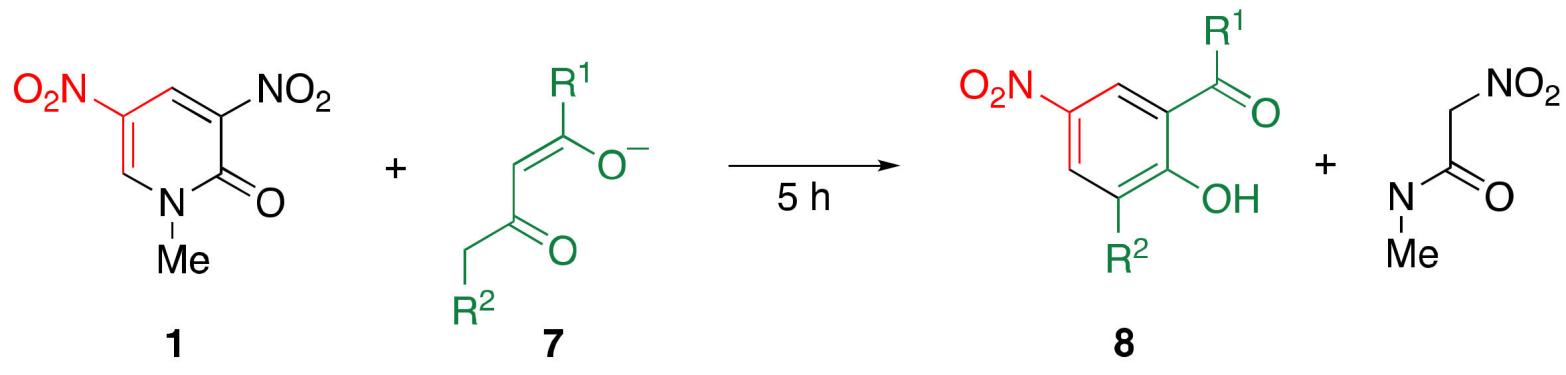
Synthesis of functionalized 4-nitrophenols by ring transformation.

R^1^	R^2^		Solv.	Temp./°C	Yield/%
OEt	COOEt	**a**	pyridine	50	91
OEt	H	**b**	pyridine	70	61
Me	H	**c**	DMF	70	53
COOEt	H	**d**	pyridine	110	42

**Table 2:**
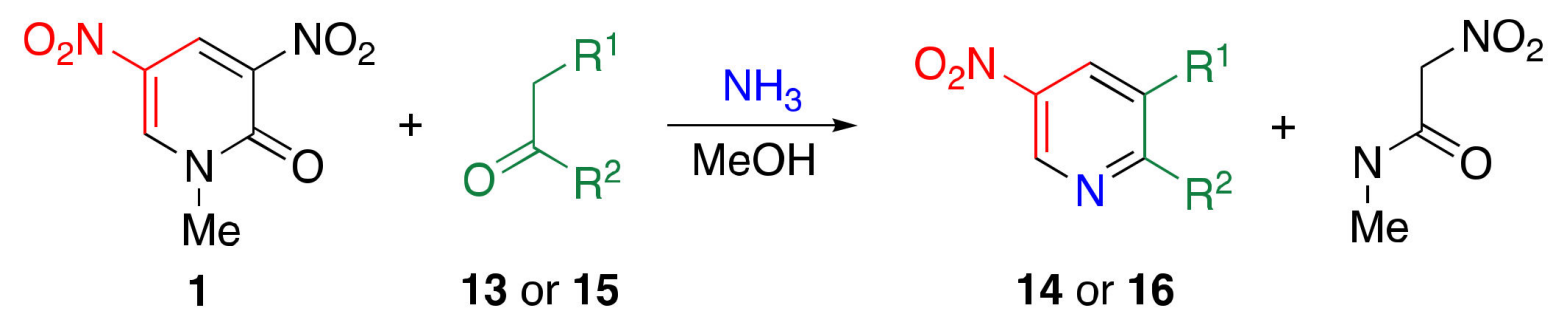
TCRT using dinitropyridine **1**, ketones, and ammonia, leading to nitropyridines.

Ketone			Condition ^1^	Product	
R^1^	R^2^				Yield/%
–(CH_2_)_4_–		**13a**	A	**14a**	83
–(CH_2_)_3_–		**13b**	A	**14b**	27
H	Ph	**15a**	A	**16a**	44
H	Ph	**15a**	B	**16a**	81
H	4-NH_2_C_6_H_4_	**15b**	B	**16b**	44
H	4-MeOC_6_H_4_	**15c**	B	**16c**	64
H	4-MeC_6_H_4_	**15d**	B	**16d**	30
H	4-NO_2_C_6_H_4_	**15e**	B	**16e**	27
H	2-pyridyl	**15f**	B	**16f**	72
H	2-furyl	**15g**	B	**16g**	62
H	2-thienyl	**15h**	B	**16h**	56
Me	Ph	**15i**	A	**16i**	10
Me	Ph	**15i**	B	**16i**	37
H	*i*-Pr	**15j**	A	**16j**	36
H	*i*-Pr	**15j**	B	**16j**	21
H	*tert*-Bu	**15k**	B	**16k**	69

^1^ Condition A: ketone (2 equiv.), ammonia (20 equiv.), heating at 70 °C for 3 h; Condition B: ketone (2 equiv.), ammonia (140 equiv.), heating at 120 °C for 3 h in an autoclave.

**Table 3:**
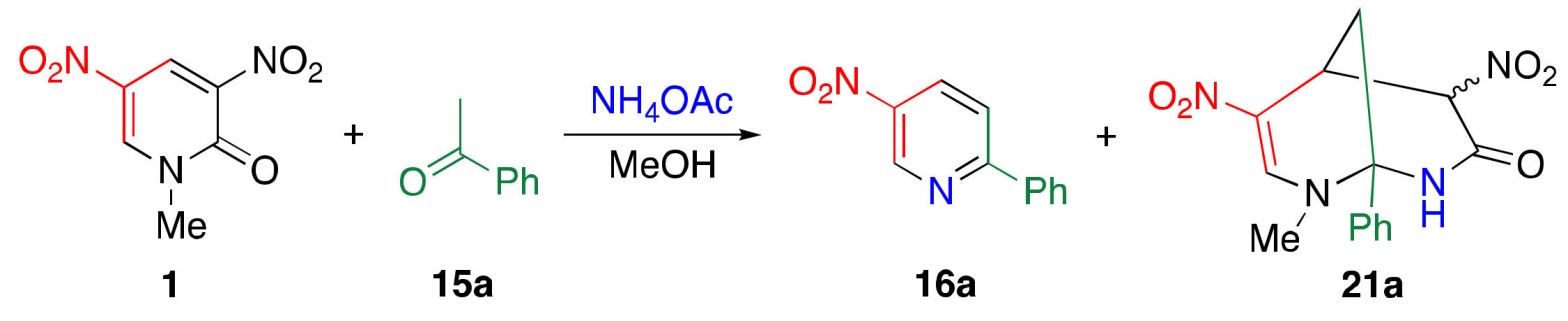
TCRT using pyridone **1** with different amounts of ammonium acetate.

NH_4_OAc /equiv.	Time/h	Yield/%		Ratio of 16a/21a	Ratio of *exo*-21a/*endo*-21a
		16a	21		
3	24	19	61	24/76	56/44
5	24	43	46	48/52	59/41
10	24	64	25	72/28	70/30
15	24	79	0	100/0	—
5 ^1^	7	92	5	95/5	60/40
15 ^1^	5	90	0	100/0	—

^1^ Microwave heating is used.

**Table 4:**
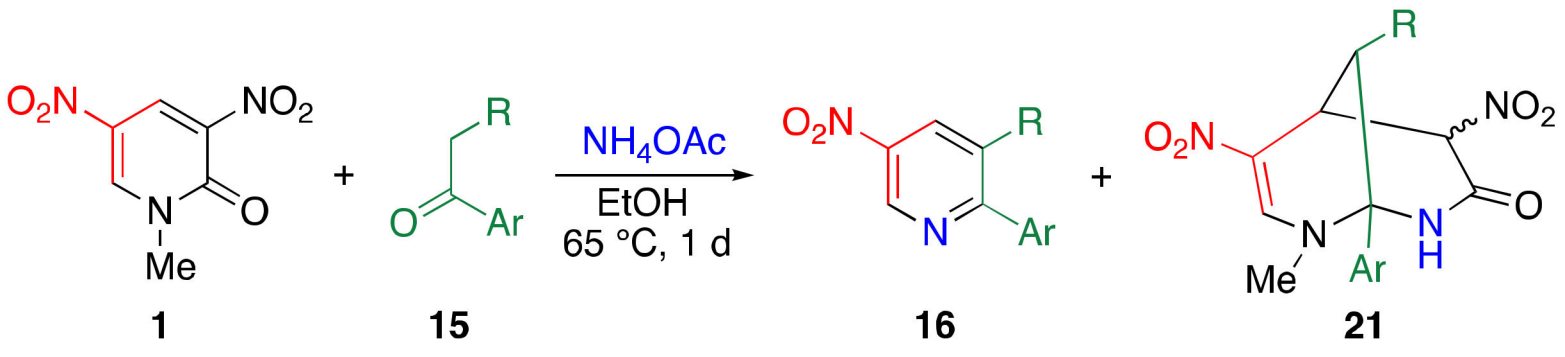
TCRT with other aromatic ketones **15**.

Ketone			NH_4_OAc/ equiv.	Yield/%		
Ar	R			16	21	16 + 21
Ph	H	**a**	15	79	0	79
4-MeOC_6_H_4_	H	**c**	5 ^1,2^	95	0	95
3-MeOC_6_H_4_	H	**l**	10	97	0	97
2-MeOC_6_H_4_	H	**m**	5	94	0	94
4-MeC_6_H_4_	H	**d**	5	88	0	88
4-ClC_6_H_4_	H	**n**	10	96	0	96
4-NO_2_C_6_H_4_	H	**e**	15	93	2	95
4-pyridyl	H	**o**	15	66	33	99
3-pyridyl	H	**p**	15	97	0	97
2-pyridyl	H	**f**	15	80	12	92
2-furyl	H	**g**	5	87	0	87
2-thienyl	H	**h**	10	85	0	95
Ph	Me	**i**	15 ^1,3^	98	0	98
Ph	Pr	**q**	15 ^1,3^	97	0	97

^1^ Microwave heating is used. ^2^ For 6 h. ^3^ At 80 °C for 2 h.

**Table 5:**
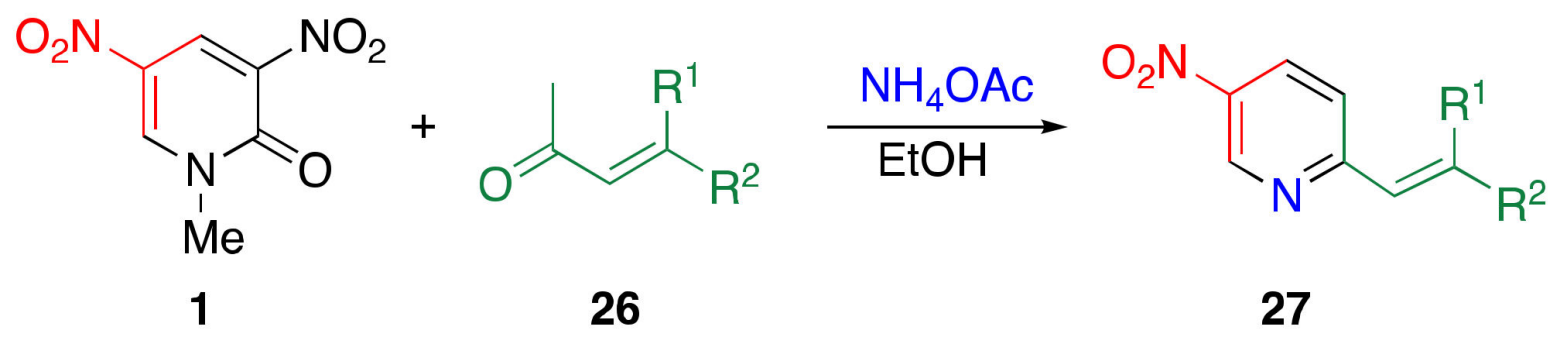
TCRT with alkenyl ketones.

Ketone			NH_4_OAc/ equiv.	Temp./°C	Time/h	Yield/%
R^1^	R^2^					
H	Ph	**a**	15	80 ^1^	4	82
H	4-MeOC_6_H_4_	**b**	30	65	24	94
H	4-ClC_6_H_4_	**c**	30	80 ^1^	4	75
H	H	**d**	30	65	24	0
Me	Me	**e**	15	80 ^1^	2	25
H	2,4,6-trimethylcyclohexyl	**f**	30	80 ^1^	6	79

^1^ Microwave heating is used.

**Table 6:**
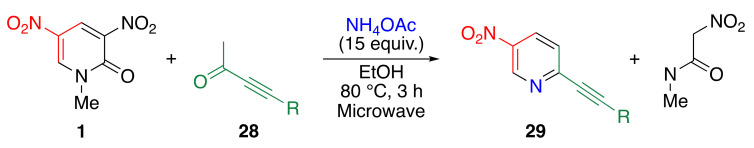
TCRT with alkynyl ketones **28**.

R		Yield/%
Ph	**a**	87
Et	**b**	80
Me_3_Si	**c**	**29c** 24/**29d** 60 ^1^

^1^ Desilylated product **29d** (R = H) is also obtained.

**Table 7:**
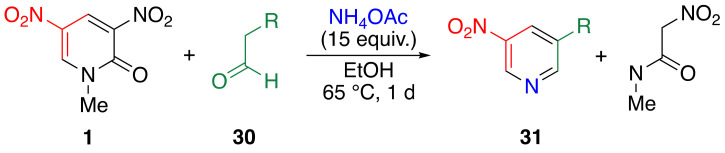
TCRT with aldehydes **30**.

R		Yield/%
Me	**a**	52
Et	**b**	86
*i*-Pr	**c**	71
*t*-Bu	**d**	68 ^1^
PhCH_2_	**e**	34
Ph	**f**	75 ^1^

^1^ Microwave heating is used.

**Table 8:**
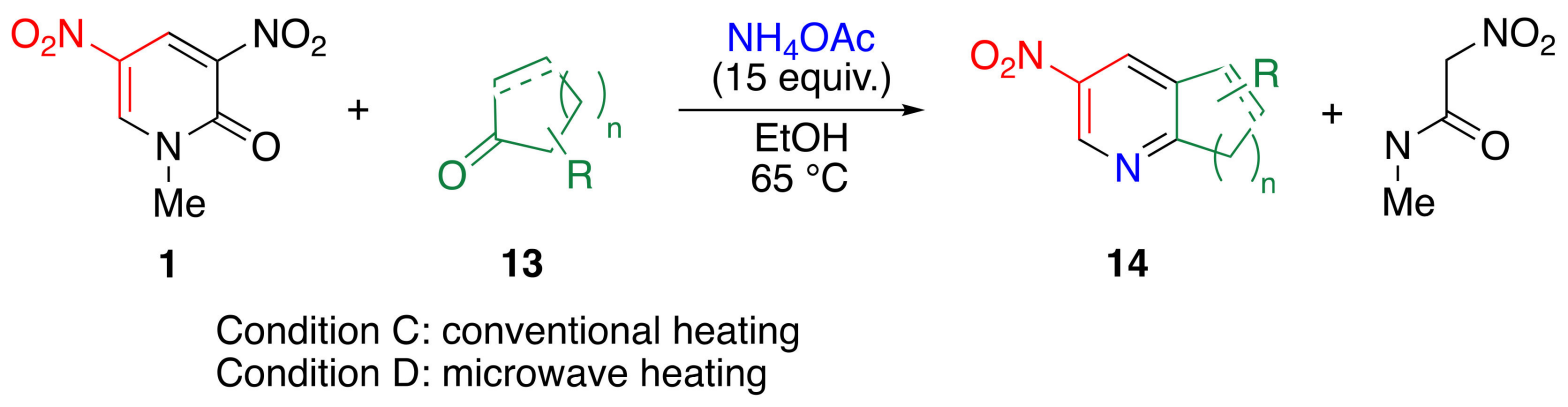
TCRT with cycloalkanones **13**.

Substrate	Product		Condition C		Condition D	
			Time/h	Yield/%	Time/h	Yield/%
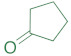	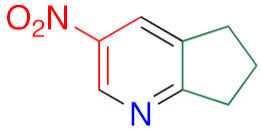	**14b**	24	67	2	87
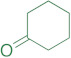	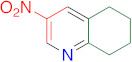	**14a**	24	95	1	97
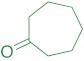	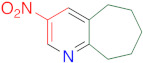	**14s**	24	94	1	91
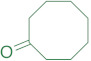	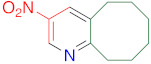	**14w**	24	85	1	95
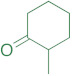	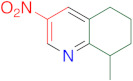	**14aa**	24	83	2	86
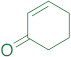	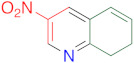	**14ab**	24	59	3	89

**Table 9:**
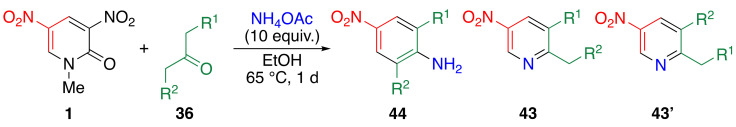
Two kinds of TCRT using aliphatic ketones **36**.

Ketone			Yield/%		
R^1^	R^2^		44	43	43’
Me	Me	**a** ^1^	50	44	—
Me	Me	**a**	83	13	—
H	H	**b**	51	47	—
Et	H	**c**	66	10	8
*i*-Pr	H	**d**	58	0	31
Pr	H	**e**	83	9	6
Et	Et	**f**	67	24	—
Pr	Pr	**g**	74	22	—
C_6_H_5_	Pr	**h**	62	24	13
C_6_H_5_	C_6_H_5_	**i**	8	81	—

^1^ Five equivalents of ammonium acetate are used.

**Table 10:**
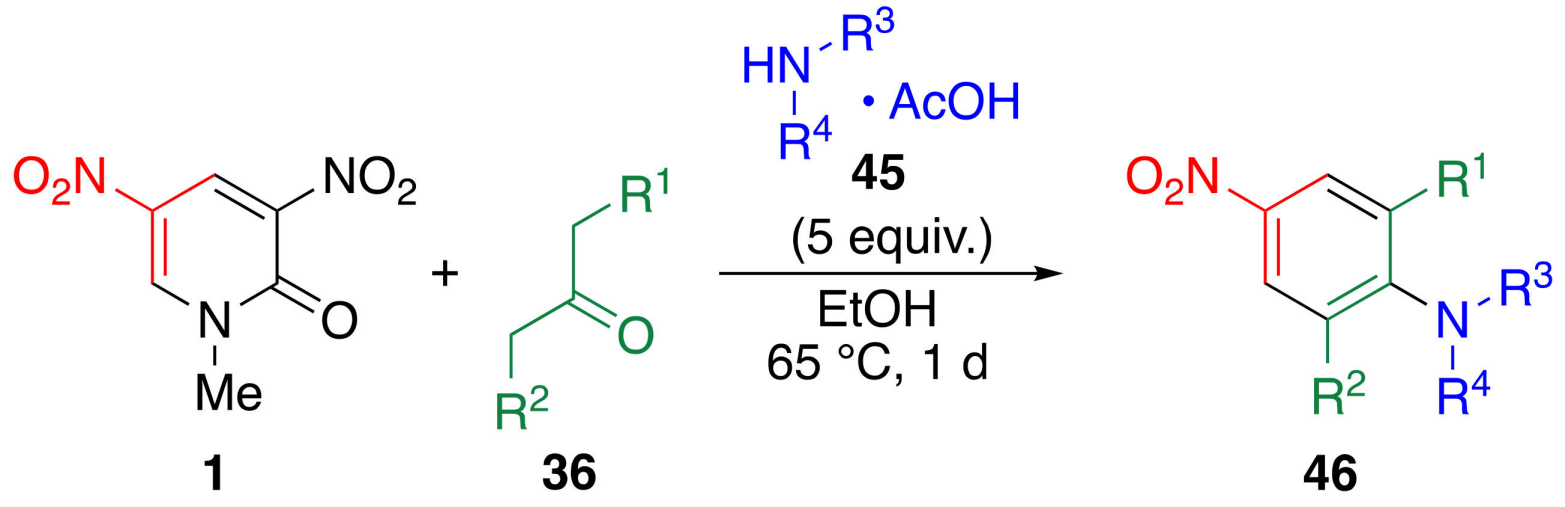
Synthesis of *N,N*,2,6-tetrasubstituted 4-nitroanilines **46** by TCRT using aliphatic ketones **36** and dialkylammonium acetate **45**.

Ketone			Amine			Product	Yield/%
R^1^	R^2^		R^3^	R^4^			
Me	Me	**36a**	Pr	H	**45A**	**46Aa**	99
Me	Me	**36a**	–(CH_2_)_4_–		**45B**	**46Ba**	98
Me	Me	**36a**	Et	Et	**45C**	**46Ca**	98
Et	H	**36c**	Pr	H	**45A**	**46Ac**	83
Et	H	**36c**	–(CH_2_)_4_–		**45B**	**46Bc**	68
Pr	H	**36e**	Pr	H	**45A**	**46Ae**	77
Pr	H	**36e**	–(CH_2_)_4_–		**45B**	**46Be**	87
Pr	H	**36e**	Et	Et	**45C**	**46Ce**	51
*i*-Pr	H	**36d**	Pr	H	**45A**	**46Ad**	83
Et	Et	**36f**	Pr	H	**45A**	**46Af**	69
Et	Et	**36f**	–(CH_2_)_4_–		**45B**	**46Bf**	68
Pr	Pr	**36g**	Pr	H	**45A**	**46Ag**	81
Pr	Pr	**36g**	–(CH_2_)_4_–		**45B**	**46Bg**	59
C_6_H_5_	Pr	**36h**	Pr	H	**45A**	**46Ah**	80
C_6_H_5_	C_6_H_5_	**36i**	Pr	H	**45A**	**46Ai**	32

**Table 11:**
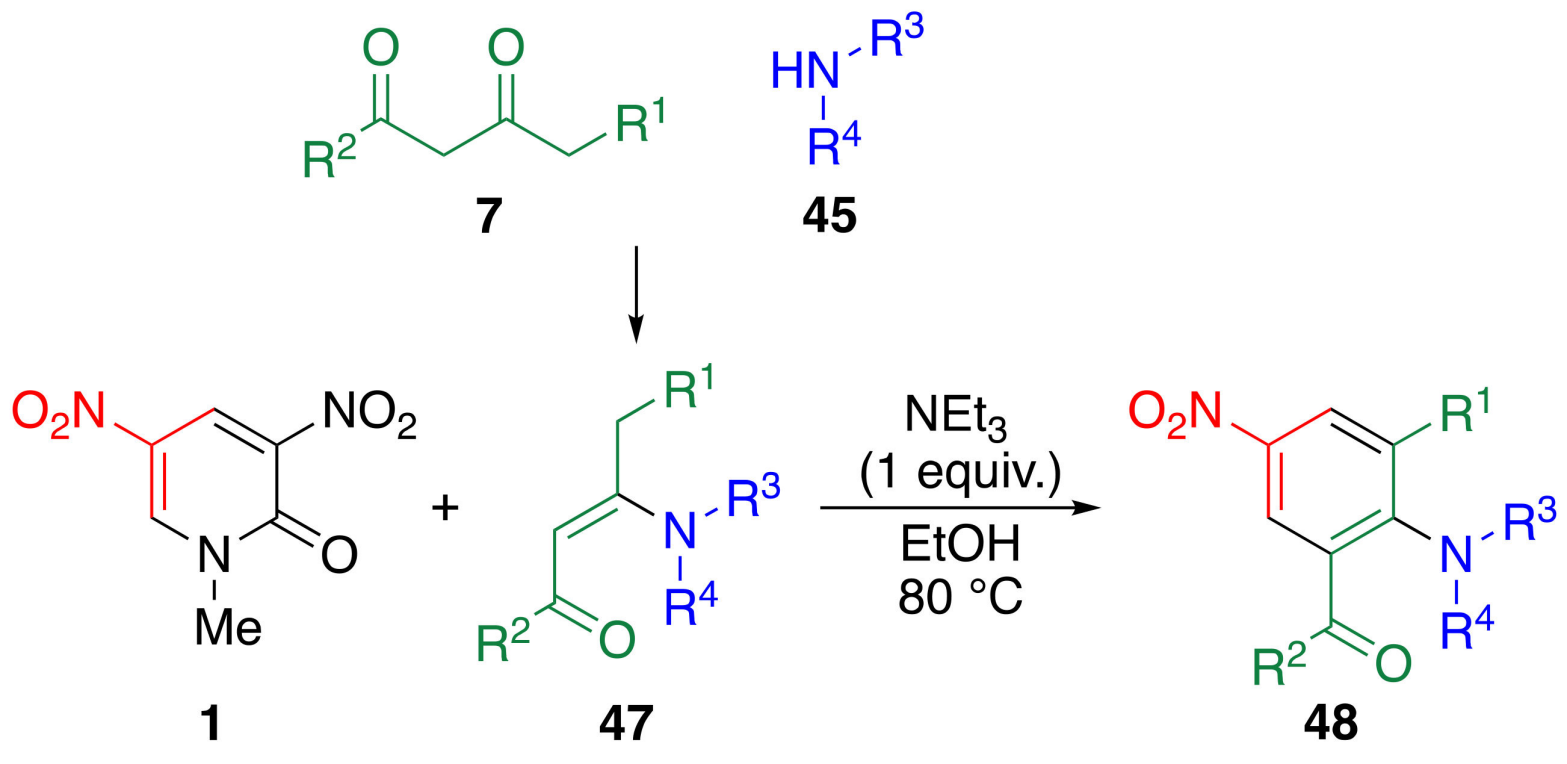
Synthesis of 2-functionalized 4-nitroanilines **48**.

1,3-Dicarbonyl Compound			Amine			Time/d	Product	Yield/%
R^1^	R^2^		R^3^	R^4^				
H	Me	**7c**	Pr	H	**45A**	2	**48Ac**	57
H	Me	**7c**	*sec*-Bu	H	**45D**	2	**48Ca**	64
H	Me	**7c**	*tert*-Bu	H	**45E**	2	**48Ca**	39
H	Me	**7c**	C_6_H_5_	H	**45F**	2	**48Ac**	23
H	Me	**7c**	4-MeC_6_H_4_	H	**45G**	2	**48Gc**	36
H	Me	**7c**	–(CH_2_)_4_–		**45B**	2	**48Bc**	87
H	C_6_H_5_	**7e**	Pr	H	**45A**	4	**48Ae**	33
H	C_6_H_5_	**7e**	Et	Et	**45C**	2	**48Ce**	45
H	OEt	**7b**	Pr	H	**45A**	1	**48Ab**	61
H	OEt	**7b**	HOCH_2_CH_2_	H	**45H**	1	**48Hb**	45
H	OEt	**7b**	Et	Et	**45C**	1	**48Cb**	57
Et	OEt	**7f**	Pr	H	**45A**	2	**48Af**	24
